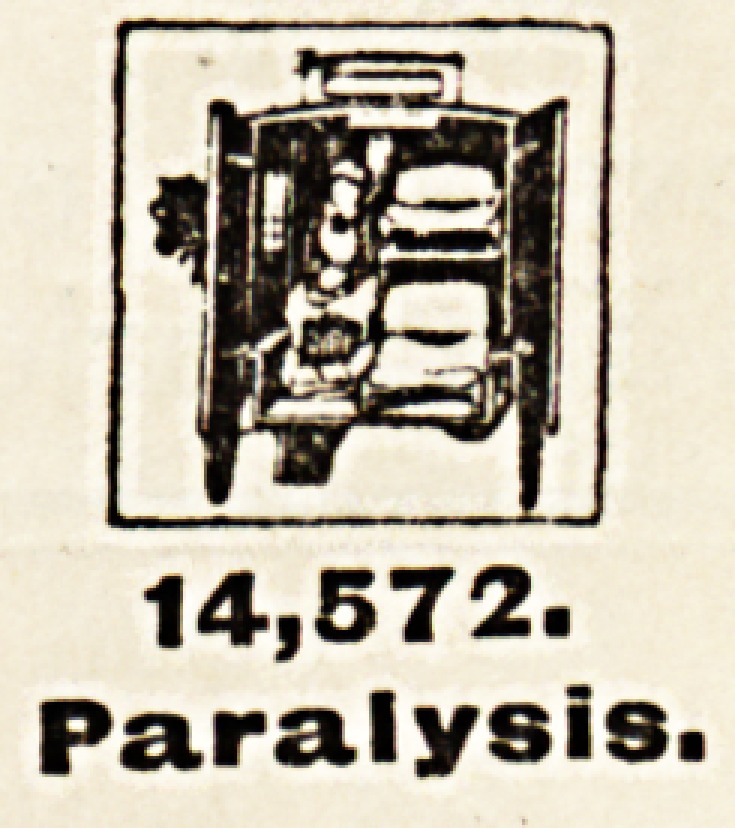# Two-And-A-Quarter Million Sufferers Helped by the Hospitals

**Published:** 1911-06-18

**Authors:** 


### Two-and-a-Quarter Million Sufferers Helped by the Hospitals.

### A SINGLE YEAR'S ROLL-CALL OF THE SICK.

In the last year for which complete figures are available, the immense total of *two million two hundred and forty-four thousand three hundred and seventy-six* patients were treated at the voluntary hospitals and dispensaries of London, the endowed hospitals of St. Bartholomew's, Guy's, and St. Thomas's, and the Infectious hospitals of the Metropolitan Asylums Board. Of these, 754,855 were men, 778,851 women, and 710,670 children. These figures only include the in-patient cases treated to a termination in the wards of the hospitals and the number of new out-patient cases treated in the out-patient departments and dispensaries, and may be taken as showing as nearly as possible the number of cases dealt with owing to the increased care taken by the authorities of our Metropolitan hospitals, under the influence of the King's and the Hospital Sunday Funds, to insure that patients shall not be counted twice in the same year at the same hospital.

**996,703. Surgical Patients.**

**Patients Suffering from Surgical Diseases.**—Of the whole number of patients received by the hospitals, *nine hundred and ninety-six thousand seven hundred and three* required surgical treatment, in addition to those treated in the special departments and hospitals for diseases of the eye, nose, throat, and ear. "Surgical" diseases include not only all accidents such as broken bones, fractured skulls, mangled limbs, and all manner of displacements and crushings of sensitive parts and organs, but also abscesses, ulcerations, cancers, and tumours of all kinds; in short all those injuries which may be produced by accident or pathological process, and which may be dealt with either by hand or instrument. It is not easy to realise that, including the special departments of our large institutions and the special hospitals, one million and a quarter patients are treated annually in the London hospitals for diseases requiring surgical treatment.

**754,455. Medical Patients.**

**Patients Suffering from Medical Diseases.**—*Seven hundred and fifty-four thousand four hundred and fifty-five* persons received medical treatment. By medical diseases are meant those diseases which are situated either as to their source and origin or in their entirety in one or the other of the three great cavities of the body. They include rheumatic fever, pneumonia, pleurisy, bronchitis, diseases of the stomach, bowels, liver, kidney, bladder, and pancreas, every kind of heart disease, many forms of brain injury, dyspepsia, constipation, most nervous diseases, and other ailments, many of them serious and many of them dangerous to life, or at least to the useful existence of the individual. Most of these diseases are out of sight; the diagnosis of their nature and extent, and the successful treatment of them, is dependent on the doctor's scientific knowledge. Remembering this, try to realise that in the hospitals of London more than three-quarters of a million persons received treatment at the hands of the foremost physicians of the day, free of cost to the patients themselves.

**180,811. Children.**

**Patients Treated at Special Hospitals for Children.**—Included in the children mentioned at the commencement of this article are *one hundred and eighty thousand eight hundred and eleven* children who were sent from homes where they could not be properly attended to for treatment in the special hospitals for the little ones.

### THE ROLL-CALL OF THE SICK.—*continued.*

172,444. Eye.

**Patients Suffering from Eye Affections.**—*One hundred and seventy-two thousand four hundred and forty-four* persons were treated in the special departments of the general hospitals or by the ophthalmic hospitals of London. It is certain that very many of these cases must have entailed terrible suffering, and many doubtless would have terminated in total loss of sight but for the skilful treatment they have received at the hospitals. Who can say how many have been saved from becoming practically helpless in the world?

95,331. Women.

**Diseases of Women and Motherhood.**—*Ninety-five thousand three hundred and thirty-one* women were treated at the Metropolitan voluntary hospitals for those diseases which are peculiar to their sex. Here it is not only our sympathy which is appealed to, but our patriotism as well. Here there is an actual demand for the payment of a debt we most justly owe. The very heart and strength of the nation lies in the home life, and the soul of the home life is the woman—the mother.

83,672. Ear and Throat.

**Patients Suffering from Diseases of the Ear, Nose, and Throat.**—At the special hospitals or special departments devoted to these diseases *eighty-three thousand six hundred and seventy-two* were treated. The affections and diseases of these organs, which are intimately connected, involve temporary and often permanent impairment of hearing, swallowing, and breathing. These functions are performed with so little effort on our part that, unless experience has taught us, it is difficult to understand what it would mean to us if we suddenly had to suffer from one or other of these affections.

60,222. Skin.

**Patients Suffering from Diseases of the Skin.**—During the year *sixty thousand two hundred and twenty-two* persons were treated for skin diseases in London. It is, perhaps, more difficult to bring home to people the claim which sufferers from skin diseases have upon their sympathy than it is in any of the other diseases which we are considering. There is not, here, as a rule, the pain, nor the danger to life, nor even such risk of permanent disablement as is the case with many of the others; but let us remember what the result would be were there no hospitals for the sufferers to go to.

43,465. Consumption.

**Patients Suffering from Consumption.**—*Forty-three thousand four hundred and sixty-five* patients suffering from phthisis or consumption were treated at the hospitals of London during the year. The very word consumption makes us afraid. There are few of us who have not seen something of its ravages, of its cruelty. Truly may consumption be called the curse of our climate. It respects neither persons nor estate, neither rich nor poor, old or young.

23,512. Fever.

**Patients Suffering from Fever.**—The number of persons who were treated for the class of fevers which are usually removed to a fever hospital was *twenty-three thousand five hundred and twelve.* This figure is, however, a misleading one, because the term fever includes much besides this class of fever. Measles, for instance, prevails in London to such an extent that more deaths occur from it than from scarlet fever. The excellent service rendered by the London Fever Hospital entitles it to the gratitude of all householders.

14,572. Paralysis.

**Patients Suffering from Paralysis and Epilepsy.**—*Fourteen thousand five hundred and seventy-two* stricken with paralysis, epilepsy, and kindred ailments received treatment at the general hospitals and at hospitals devoted to these maladies. To workers busy with hand and brain these sufferers must particularly appeal. It is impossible to dissociate nervous breakdown from the toil and hurry of existence, especially in a vast centre like London. It is appalling to think that at any moment any one of us may be struck down, perhaps without the slightest warning. No disease is more sudden than paralysis, surely none more pitiful.

So this great army of sufferers, numbering over two and a quarter millions, claims our sympathy and our help year by year. To the strong, to those in health who are able to provide for those dependent upon them, to those who know what ill-health means, who have suffered from disease of one kind or another, and who, either in the hospital or under the skill and care of the doctors and nurses trained in the hospitals, have been restored to health and usefulness, we confidently appeal on behalf of the London hospitals.

### THE ROLL-CALL OF THE SICK.

| | | | |
|---|---|---|---|
| Sufferers needing Surgical Aid | 996,703 | Sufferers from Skin Diseases | 60,222 |
| Sufferers needing Medical Care | 754,455 | Consumptives | 43,465 |
| Sufferers from Eye Troubles | 172,444 | Fever Patients | 23,512 |
| Diseases of Women | 95,331 | Paralysis and Epilepsy | 14,572 |
| Diseases of the Ear, Nose, and Throat | 83,672 | Total | 2,244,376 |

## Figures and Tables

**Figure f1:**
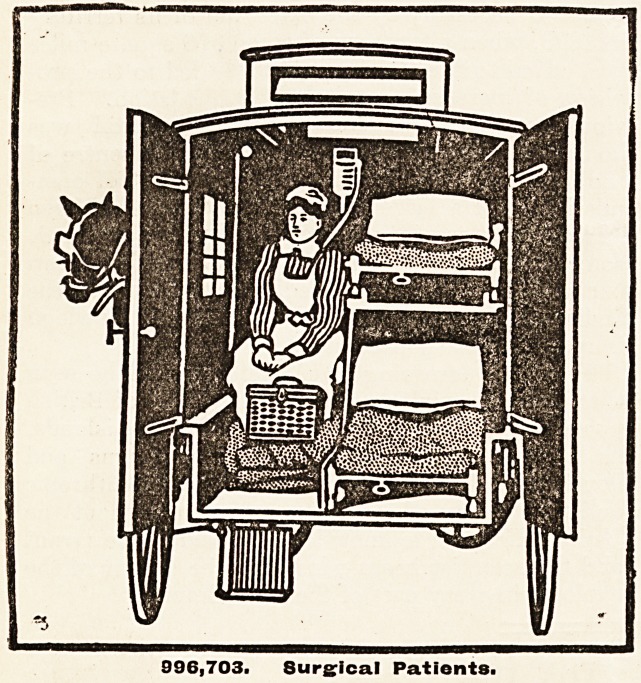


**Figure f2:**
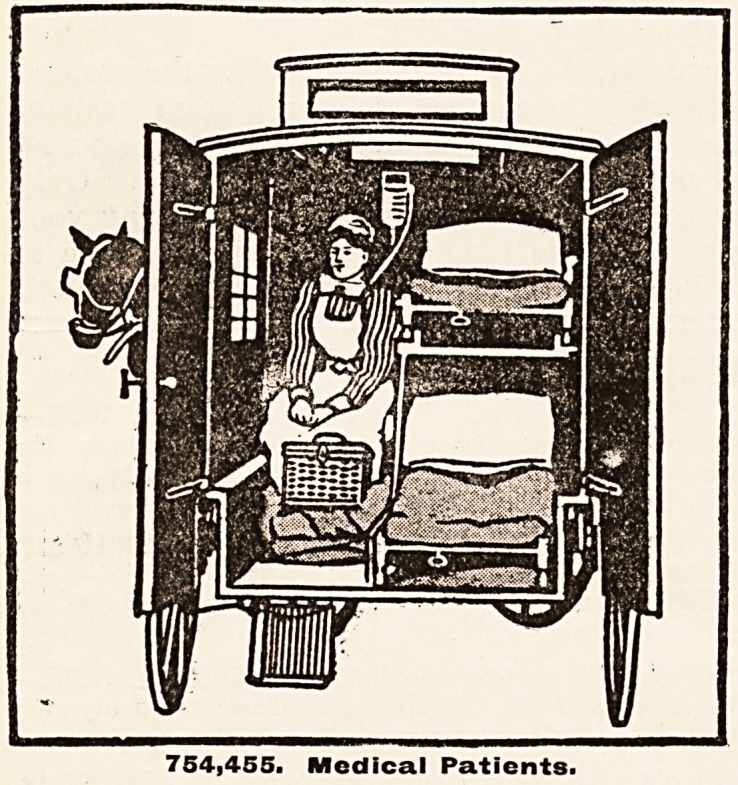


**Figure f3:**
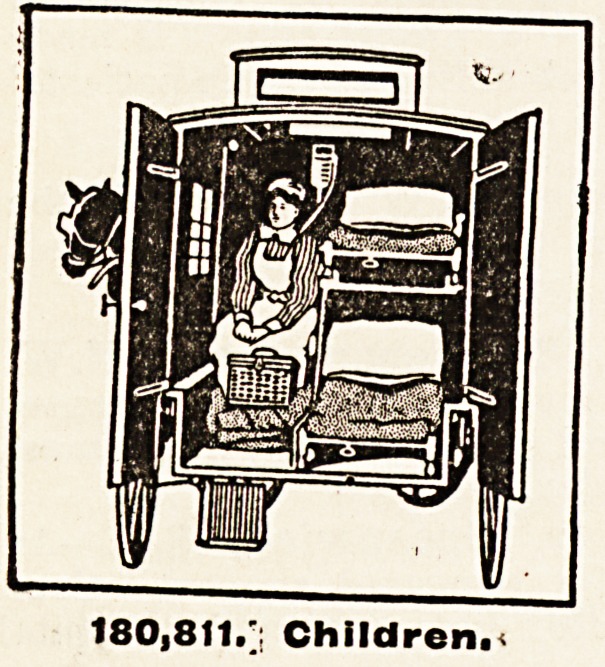


**Figure f4:**
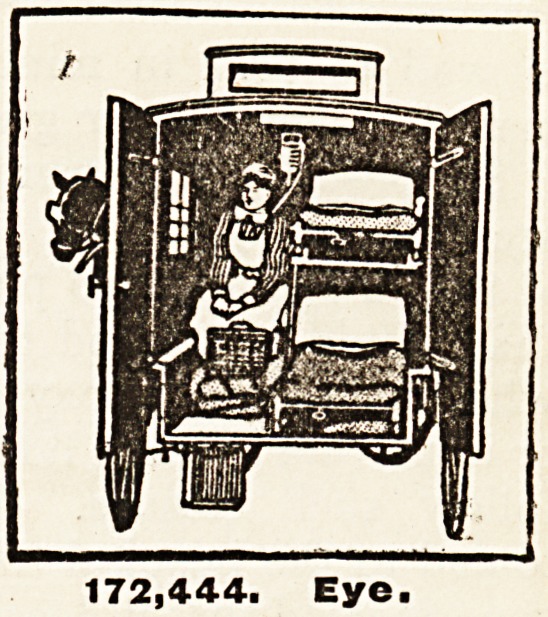


**Figure f5:**
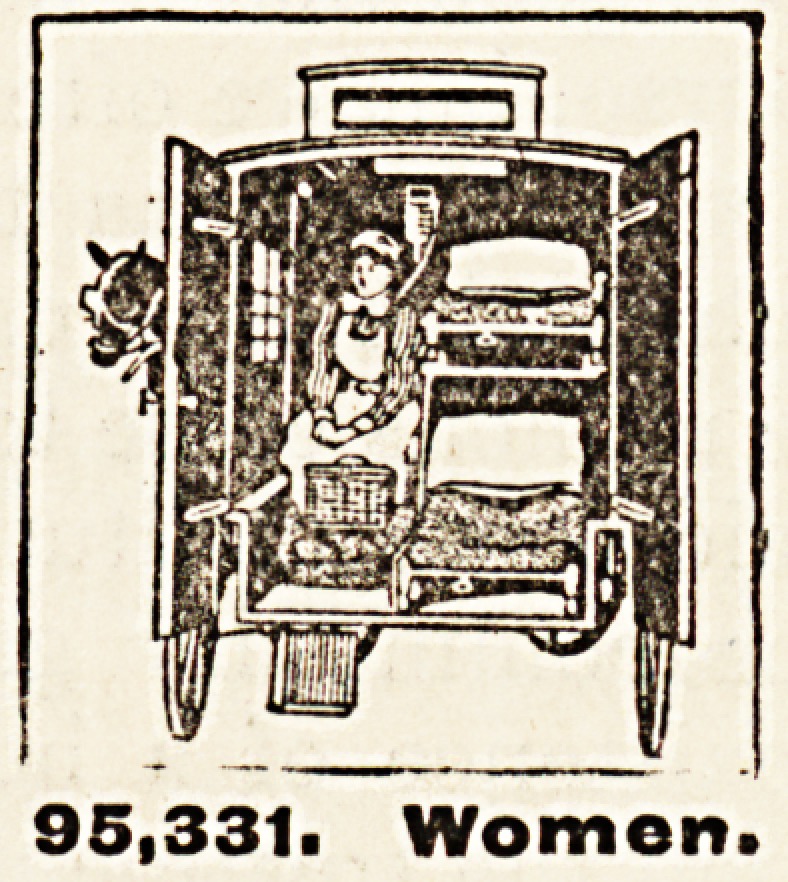


**Figure f6:**
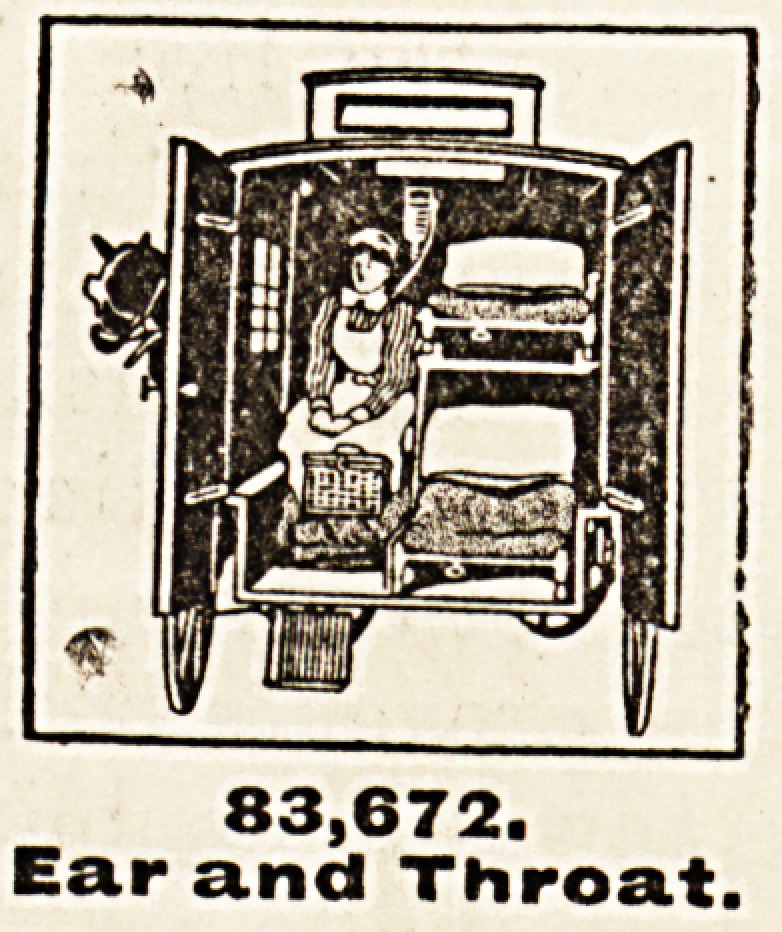


**Figure f7:**
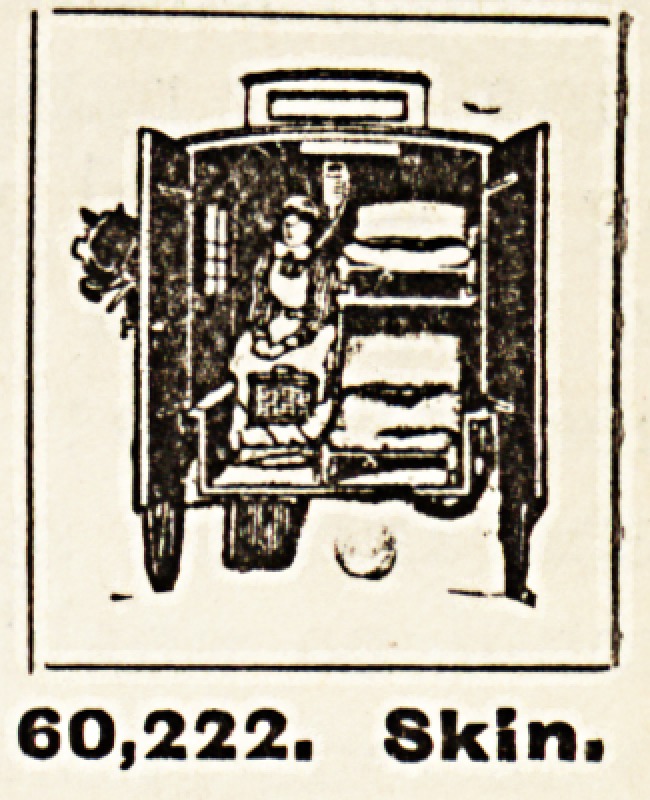


**Figure f8:**
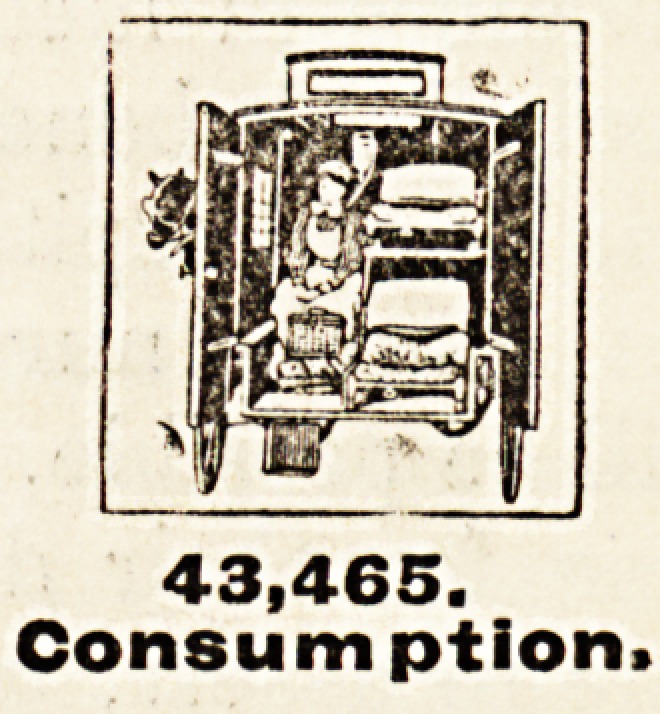


**Figure f9:**
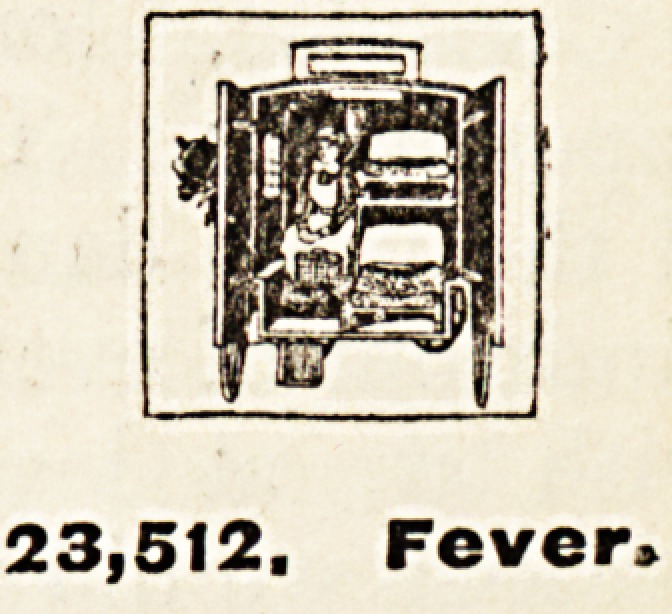


**Figure f10:**